# Prognostic value of serum high‐density lipoprotein cholesterol elevation in nonsmall cell lung cancer patients receiving radical surgery

**DOI:** 10.1002/ctm2.94

**Published:** 2020-06-05

**Authors:** Fan Luo, Kang‐mei Zeng, Zhong‐han Zhang, Ting Zhou, Jian‐hua Zhan, Fei‐teng Lu, Yun‐peng Yang, Yan Huang, Li Zhang, Hong‐yun Zhao

**Affiliations:** ^1^ State Key Laboratory of Oncology in South China Collaborative Innovation Center for Cancer Medicine Guangdong Esophageal Cancer Institute Sun Yat‐sen University Cancer Center Guangzhou P. R. China

Dear Editor,

The prognosis for nonsmall cell lung cancer (NSCLC) patients after accepting radical resection has been reported heterogeneously.^1^, ^2^, ^3^ In recent years, the evaluation of prognosis has been developed at molecular levels, largely expanding the predictive power.^4^, ^5^, ^6^, ^7^ Unfortunately, these measurements based on molecular markers are of high cost, complicated to analyze and limited in facilities.^8^ Therefore, it is of paramount importance to explore simple and cost‐efficient approaches to better predict prognosis of NSCLC patients. We performed the retrospective investigation to identify the prognostic value of pretreatment lipids and dynamical lipid alterations for relapse in NSCLC patients after radical surgery.

This retrospective study involved 103 patients and the median clinical follow‐up time was 59.6 months. Distribution according to the clinical characteristics of all patients was described in Table S1. In this study, fasting serum lipids, including cholesterol, triglyceride, low‐density lipoprotein cholesterol (LDL‐C), apolipoprotein‐B (ApoB), high‐density lipoprotein cholesterol (HDL‐C), and apolipoprotein A‐I (ApoA‐I), were tested before surgery and at 6‐month follow‐up after surgery in limited‐stage NSCLC patients. The paired *t*‐test analysis was applied to conduct a comparison of the aforementioned serum lipids at two different time points, and we observed significant elevation of various lipids and lipoproteins in postoperative NSCLC patients at 6‐month follow‐up (*P ≤* .001, Table S2).

To further determine the correlation between baseline lipid levels and the prognosis of postoperative NSCLC patients, we used X‐tile program to determine the optimal cutoff values first, and the X‐tile analysis yielded 1.25, 1.84, and 0.62 mmol/L of baseline HDL‐C (Figure 1A‐C), LDL‐C (Figure 1D‐F), and ApoB (Figure 1G‐I), respectively, as the optimal cutoff values for high/low levels of the aforementioned lipids. As shown in Table S3, patients with lower baseline HDL‐C, higher baseline LDL‐C, or ApoB have longer disease‐free survival (DFS). Furthermore, Kaplan‐Meier analysis revealed that the DFS for patients with baseline HDL‐C less than 1.25 mmol/L was significantly longer compared with those with baseline HDL‐C level higher than 1.25 mmol/L (58.5% vs 23.8%, *P *= .017, Figure 2A). However, patients with baseline LDL‐C level above 1.84 mmol/L and the baseline ApoB level exceeding 0.62 mmol/L presented better DFS than those with baseline LDL‐C and ApoB level below their respective cut‐off values (56.8% vs 31.8%, *P *= .022 for LDL‐C, Figure 2B; 54.3% vs 22.2%, *P *= .032 for ApoB, Figure 2C).

**Figure 1:**
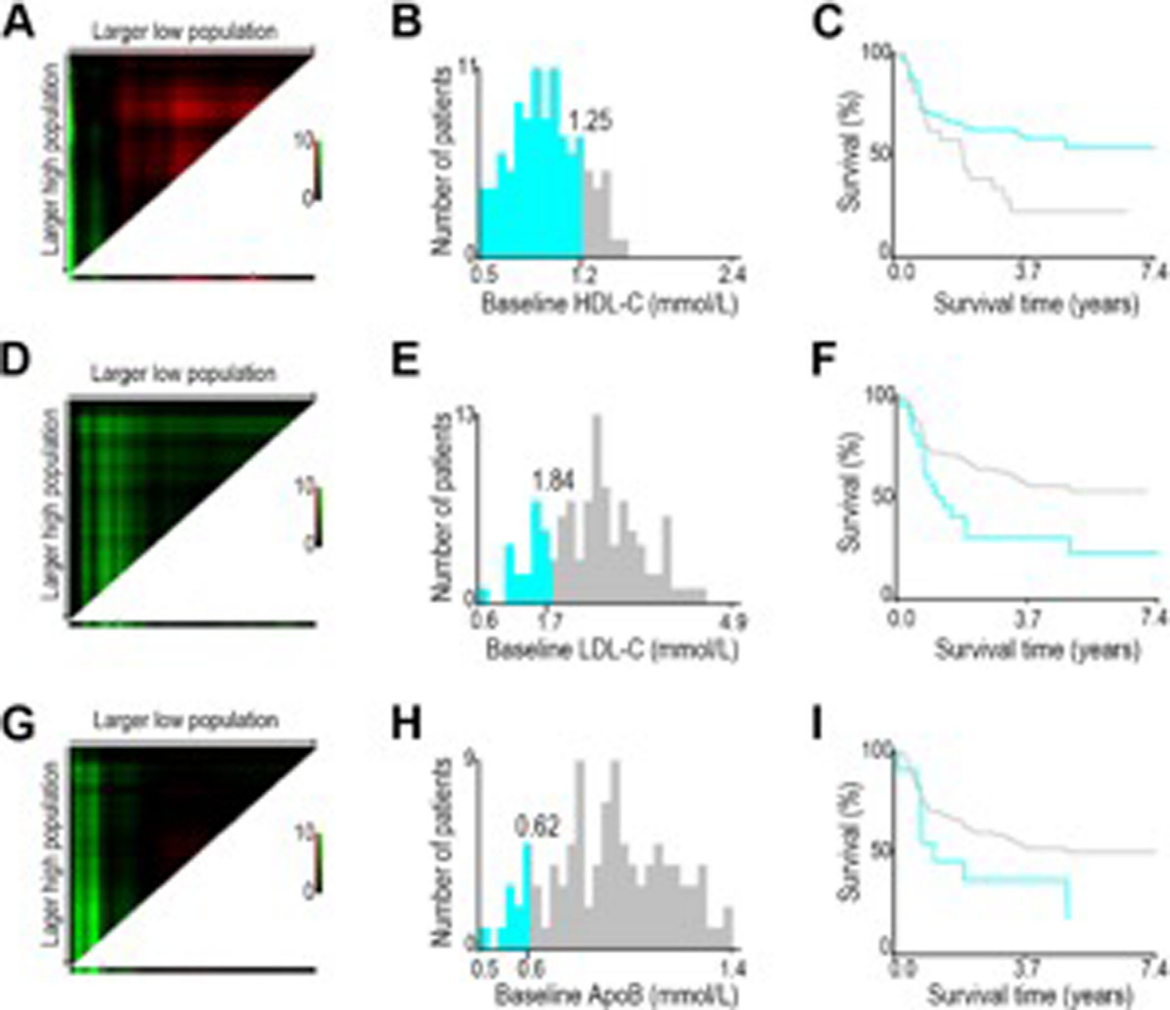
X‐tile analysis of total risk score and survival graphs stratified by the optimal cutoff values calculated by the levels of serum HDL‐C (A‐C), LDL‐C (D‐F) and ApoB (G‐I)

**Figure 2:**
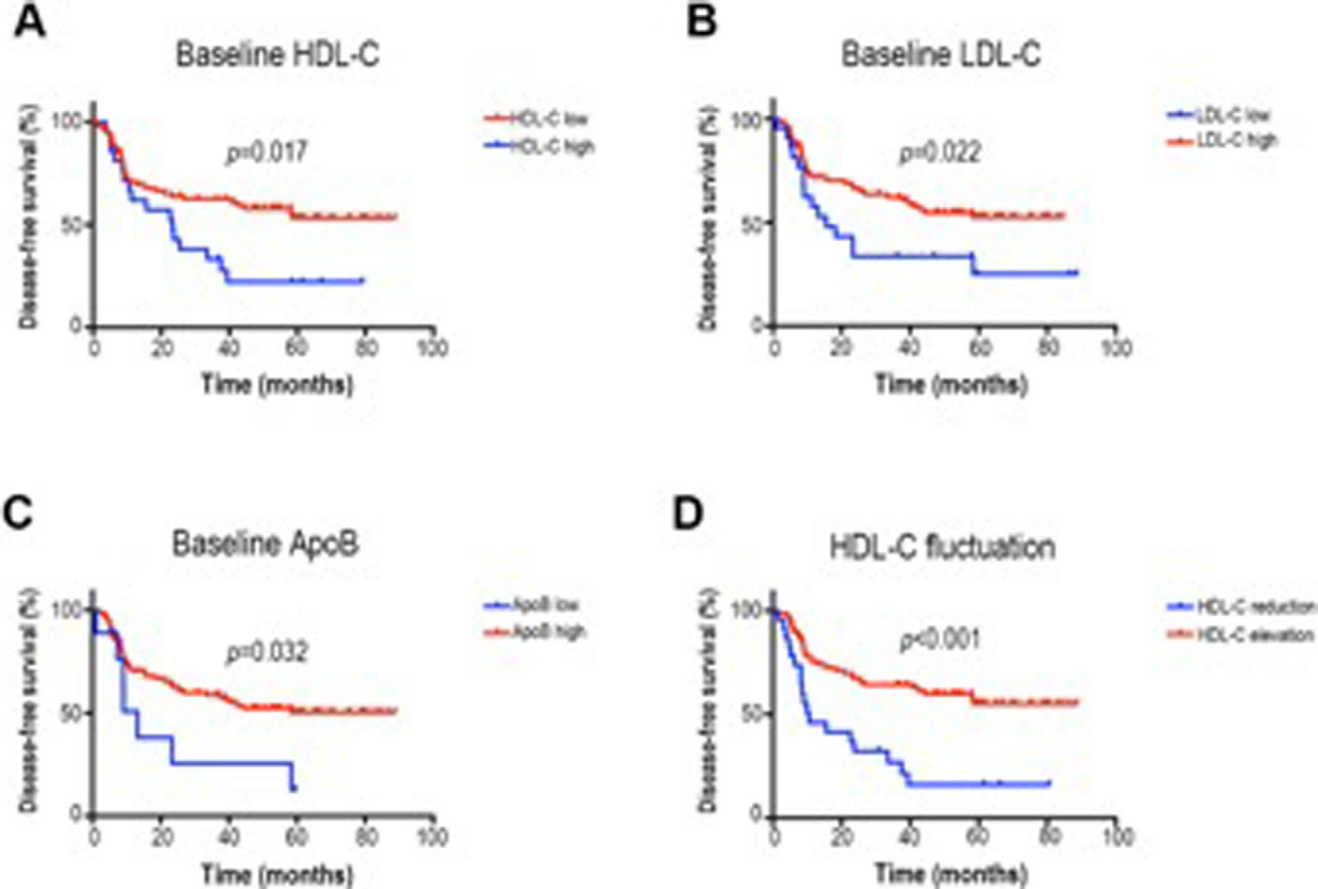
The survival curves of baseline serum HDL‐C, LDL‐C, and ApoB stratified by the cutoff values calculated by the X‐tile and the survival curve stratified by HDL‐C elevation and reduction. A, DFS was statistically significant between patients with baseline serum HDL‐C less than 1.25 mmol/L and those with HDL‐C higher than 1.25 mmol/L (58.5% vs 23.8%, *P* = .017). B, DFS was statistically different between patients with baseline LDL‐C level above 1.84 mmol/L and those with LDL‐C level below 1.854 mmol/L (56.8% vs 31.8%, *P* = .022). C, DFS was statistically significant between patients with baseline ApoB exceeding 0.62 mmol/L and those with ApoB level less than 0.62 mmol/L (54.3% vs 22.2%, *P* = .032). D, DFS was statistically significant between patients with serum HDL‐C elevation and those with serum HDL‐C reduction at six follow‐up compared to baseline (60.0% vs 21.7%, *P* < .001)

Considering the strong correlation between dynamic elevations of serum lipids and better response of chemotherapy in lymphoma patients reported previously,^9^ we further investigated whether the dynamical lipid fluctuations could exist in NSCLC patients after radical surgery and further influence their disease relapse. As demonstrated in Table S4, dynamical HDL‐C elevation exhibited significant association with DFS. Patients with HDL‐C increasing at the 6‐month follow‐up exhibited superior DFS (60.0% vs 21.7%, *P *< .001, Figure 2D). Moreover, for DFS, the predictive value of HDL‐C elevation at six‐month follow‐up was revealed in the univariate cox model (reduction vs elevation, hazard ratio (HR), 95% CI, 1.00 (ref.) vs 0.335 (0.187‐0.600), *P <* .001) and was also observed in the multivariate cox analysis (reduction vs elevation, HR, 95% CI, 1.00 (ref.) vs 0.333 (0.154‐0.717), *P *= .005). Nevertheless, the positive predictive ability of baseline lower HDL‐C, higher ApoB, and LDL‐C levels was merely manifested in univariate cox analysis for DFS (Table S5).

In general, our study found a marked correlation between the serum lipid profile and DFS in patients with radically resected NSCLC for the first time. Patients with lower HDL‐C, higher LDL‐C, or Apo‐B level at baseline are prone to have longer DFS. Furthermore, elevation of HDL‐C level was a favorable prognostic index for DFS in patients with NSCLC receiving radical surgery. Moreover, both univariate and multivariate analyses indicated that the HDL‐C elevation at 6‐month follow‐up could independently help identifying patients susceptible to early cancer recurrence. These findings are novel and of remarkable significance to encourage further study in clinic. And the specific implication of HDL‐C elevation in the prognosis of NSCLC patients will merely be illuminated by prospective studies, involving large, well‐designed populations along with functional validation in vitro and in vivo.

## CONFLICT OF INTEREST

All the authors declare no conflict of interest.

## Supporting information

Supplementary materials

## Data Availability

All the data and materials are available upon reasonable request from the corresponding author.
