# Supplementary material for: Prognostic value of serum high‐density lipoprotein cholesterol elevation in nonsmall cell lung cancer patients receiving radical surgery
Source: Clin Transl Med. 2020 Jun 5;10(2):e94. doi: 10.1002/ctm2.94 (PMC7403695; doi:10.1002/ctm2.94)
Supplement: Supplementary file 1 — Supplementary materials [file CTM2-10-e94-s001.docx]

**Supplementary materials**

**Table S1. Characteristics of All Patients**

| **Characteristics** | **Cases(n=103)** | **Percentage (%)** |
| --- | --- | --- |
| **Age(years)** |  |  |
| Median(range) | 60.7 (22–83) | |
| ＜65 | 66 | 64.1 |
| ≥65 | 37 | 35.9 |
| **Gender** |  |  |
| Male | 70 | 68 |
| Female | 33 | 32 |
| **BMI** |  |  |
| Median(range) | 22.7 (16.7–31.2) |  |
| <24 kg/m^2^ | 69 | 67 |
| ≥24 kg/m^2^ | 34 | 33 |
| **Histological** |  |  |
| Adenocarcinoma | 50 | 48.5 |
| Non-adenocarcinoma | 53 | 51.5 |
| **Smoking status** |  |  |
| Non-smokers | 42 | 40.8 |
| Smokers | 61 | 59.2 |
| **Drinking status** |  |  |
| Non-drinkers | 69 | 67 |
| Drinkers | 34 | 33 |
| **Diagnosis of diabetes** |  |  |
| Non-diabetics | 93 | 90.3 |
| Diabetics | 10 | 9.7 |
| **Diagnosis of hypertension** |  |  |
| Non-hyperpietics | 87 | 84.5 |
| Hyperpietics | 16 | 15.5 |
| **Disease stage** |  |  |
| I | 21 | 20.4 |
| II-III | 82 | 79.6 |
| **Radiotherapy** |  |  |
| 0 | 100 | 97.1 |
| 1 | 3 | 2.9 |

**Abbreviations:** BMI = Body Mass Index.

**Table S2: The alterations of lipids in all patients**

| Lipids | Mean± SD | P valueф |
| --- | --- | --- |
| **Cholesterol (mmol/L)** |  | **< 0.001** |
| Level at diagnosis | 4.22±0.95 | |
| Level at follow-up | 5.10±1.09 |  |
| Differenceƪ | 0.88±1.06 |  |
| **Triglyceride (mmol/L)** |  | **0.001** |
| Level at diagnosis | 1.32±0.62 |  |
| Level at follow-up | 1.68±1.26 |  |
| Differenceƪ | 0.36±1.08 |  |
| **HDL-C (mmol/L)** |  | **< 0.001** |
| Level at diagnosis | 1.00±0.30 |  |
| Level at follow-up | 1.28±0.35 |  |
| Differenceƪ | 0.28±0.39 |  |
| **LDL-C (mmol/L)** |  | **< 0.001** |
| Level at diagnosis | 2.55±0.83 |  |
| Level at follow-up | 3.14±0.89 |  |
| Differenceƪ | 0.59±0.96 |  |
| **ApoA-I (g/L)** |  | **< 0.001** |
| Level at diagnosis | 0.98±0.19 |  |
| Level at follow-up | 1.36±0.26 |  |
| Differenceƪ | 0.38±0.28 |  |
| **ApoB (g/L)** |  | **< 0.001** |
| Level at diagnosis | 0.92±0.21 |  |
| Level at follow-up | 1.01±0.25 |  |
| Differenceƪ | 0.09±0.22 |  |

Abbreviations: SD= standard deviations, HDL-C = high-density lipoprotein cholesterol, LDL-C = low-density lipoprotein cholesterol, ApoA-I = apolipoprotein A-I, ApoB = apolipoprotein B. Data are mean ± standard deviations. ƪDifference = lipidsfollow-up. -lipidsat diagnosis фCompared with paired t-test.

**Table S3: The baseline lipids for DFS**

| Lipids | N (%) | DFS (%) | P value |
| --- | --- | --- | --- |
| **Cholesterol ^#^** |  |  | 0.075 |
| High | 78 55.1 | |  |
| Low | 25 | 40.0 |  |
| **Triglyceride ^#^** |  |  | 0.232 |
| High | 76 | 55.3 |  |
| Low | 27 | 40.7 |  |
| **HDL-C ^#^** |  |  | **0.017** |
| High | 21 | 23.8 |  |
| Low | 82 | 58.5 |  |
| **LDL-C ^#^** |  |  | **0.022** |
| High | 81 | 56.8 |  |
| Low | 22 | 31.8 |  |
| **ApoA-I ^#^** |  |  | 0.262 |
| High | 49 | 57.1 |  |
| Low | 54 | 46.3 |  |
| **ApoB ^#^** |  |  | **0.032** |
| High | 94 | 54.3 |  |
| Low | 9 | 22.2 |  |

Abbreviations: DFS= disease free survival. **^#^** Comparison between baseline lipids of high and low.

**Table S4: The lipids alterations for DFS**

| Lipids | N (%) | DFS (%) | P value |
| --- | --- | --- | --- |
| **Cholesterol** **elevation^#^** |  |  | 0.213 |
| Yes | 84 53.6 | |  |
| No | 19 | 42.1 |  |
| **Triglyceride elevation^#^** |  |  | 0.349 |
| Yes | 69 | 47.8 |  |
| No | 34 | 58.8 |  |
| **HDL-C elevation^#^** |  |  | **< 0.001** |
| Yes | 80 | 60.0 |  |
| No | 23 | 21.7 |  |
| **LDL-C elevation^#^** |  |  | 0.752 |
| Yes | 79 | 51.9 |  |
| No | 24 | 50.0 |  |
| **ApoA-I elevation^#^** |  |  | 0.925 |
| Yes | 96 | 51.0 |  |
| No | 7 | 57.1 |  |
| **ApoB elevation^#^** |  |  | 0.387 |
| Yes | 68 | 52.9 |  |
| No | 35 | 48.6 |  |

Abbreviations: DFS= disease free survival. **^#^** Comparision between lipidsfollow-up and lipidsat diagnoses

**Table S5: Predictive factors for DFS by univariate and multivariate analysis**

| **Univariate analyses** **Multivariate analyses** | | | | |
| --- | --- | --- | --- | --- |
|  | **HR (95% CI)** | **P value** | **HR (95% CI)** | **P value** |
| **Gender** |  |  |  |  |
| Female | 1.000(ref.) | | 1.000(ref.) |  |
| Male | 1.584(0.827-3.302) | 0.165 | 2.133(0.804-5.662) | 0.128 |
| **Year** |  |  |  |  |
| ≥65 | 1.000(ref.) |  | 1.000(ref.) |  |
| <65 | 0.616(0.352-1.077) | 0.089 | 0.899(0.471-1.713) | 0.746 |
| **BMI** |  |  |  |  |
| ≥24 kg/m^2^ | 1.000(ref.) |  | 1.000(ref.) |  |
| <24 kg/m^2^ | 1.073(0.592-1.944) | 0.817 | 1.236(0.600-2.544) | 0.565 |
| **Histological** |  |  |  |  |
| Adenocarcinoma | 1.000(ref.) |  | 1.000(ref.) |  |
| Non-adenocarcinoma | 1.742(0.987-3.073) | 0.055 | 1.930(0.983-3.790) | 0.056 |
| **Disease Stage** |  |  |  |  |
| II-III | 1.000(ref.) |  | 1.000(ref.) |  |
| I | 0.685(0.332-1.412) | 0.305 | 0.889(0.403-1.961) | 0.771 |
| **Smoking status** |  |  |  |  |
| Smokers | 1.000(ref.) |  | 1.000(ref.) |  |
| Non-smokers | 0.789(0.442-1.407) | 0.422 | 2.325(0.914-5.913) | 0.076 |
| **Drinking status** |  |  |  |  |
| Drinkers | 1.000(ref.) |  | 1.000(ref.) |  |
| Non-drinkers | 0.839(0.467-1.507) | 0.556 | 0.899(0.447-1.806) | 0.764 |
| **Baseline HDL-C** |  |  |  |  |
| High | 1.000(ref.) |  | 1.000(ref.) |  |
| Low | 0.490(0.269-0.891) | **0.019** | 0.556(0.240-1.292) | 0.173 |
| **Baseline LDL-C** |  |  |  |  |
| Low | 1.000(ref.) |  | 1.000(ref.) |  |
| High | 0.499(0.272-0.917) | **0.025** | 0.492(0.215-1.129) | 0.094 |
| **Baseline ApoB** |  |  |  |  |
| Low | 1.000(ref.) |  | 1.000(ref.) |  |
| High | 0.427(0.191-0.954) | **0.038** | 0.825(0.265-2.565) | 0.739 |
| **HDL-C elevation** |  |  |  |  |
| No | 1.000(ref.) |  | 1.000(ref.) |  |
| Yes | 0.335(0.187-0.600) | **<0.001** | 0.333(0.154-0.717) | **0.005** |

Abbreviations: DFS=Disease Free Survival; HR=Hazard ratio; CI=Confidence interval.
